# Short‐Term Oral Spermidine Supplementation Modifies Aspects of Neurodegenerative Disease in Flies and Mice With MPS III

**DOI:** 10.1002/jimd.70195

**Published:** 2026-04-28

**Authors:** Helen Beard, Sonia Dayan, Karissa Barthelson, Laura Hewson, Louise V. O'Keefe, Kim M. Hemsley

**Affiliations:** ^1^ Childhood Dementia Research Group, Flinders Health and Medical Research Institute, College of Medicine and Public Health, Flinders University Bedford Park South Australia Australia; ^2^ Department of Molecular and Biomedical Science School of Biological Sciences, the University of Adelaide Adelaide South Australia Australia

## Abstract

Mucopolysaccharidosis type III (MPS III) is a group of autosomal recessive neurodegenerative lysosomal storage disorders that causes progressive cognitive and physical impairment, predominantly in child/early adulthood. The median age of death is 17 years as there is no safe, effective treatment approved. Using faithful *Drosophila* and murine models of MPS III, we have characterised the MPS IIIA and MPS IIIC fly metabolome, explored the ability of oral spermidine supplementation to ameliorate clinical disease in the fly models and explored its mechanism of action in MPS IIIA mice. Spermidine is a polyamine naturally synthesised by the body. Its manufacture decreases with age. Supplementation has been reported to stimulate autophagy, reduce cell senescence and increase health/lifespan. The metabolomic evaluation confirmed that whole MPS IIIA and MPS IIIC flies exhibit a progressively deranged metabolome. Significantly up‐regulated metabolites were those involved in nucleotide and purine metabolism. The most significantly down‐regulated metabolites were those involved in ascorbate and aldarate metabolism. Further, spermidine levels decreased significantly in all fly genotypes with age. In short‐term studies, food enriched with 5 mM spermidine improved overall fly activity and climbing ability. A 4‐week study in pre‐symptomatic MPS IIIA mice (3‐ or 6‐mM spermidine, supplemented in drinking water) revealed no improvement in microgliosis or lysosomal compartment size; however, we observed a significant reduction in the astroglial response in the brain, which is believed to drive disease progression. Longer‐term confirmatory studies in larger cohorts of MPS III animals are now warranted to determine whether spermidine supplementation is of benefit in preventing or slowing clinical disease in this and other childhood dementias.

## 1 Introduction

MPS III is a group of autosomal recessive neurodegenerative lysosomal storage disorders caused by mutations in genes encoding heparan sulfate degrading enzymes, resulting in insufficient lysosomal enzyme/protein synthesis and accumulation of partially degraded heparan sulfate fragments intracellularly in tissues and bodily fluids. There are four subtypes (MPS IIIA, B, C and D) based on the identity of the gene/lysosomal protein impacted (N‐sulfoglucosamine sulfohydrolase (SGSH; MPS IIIA [1, 2])), N‐acetyl‐α‐glucosaminidase (NAGLU; MPS IIIB [3, 4]), acetyl‐CoA: α‐glucosaminide N‐acetyltransferase (HGSNAT; MPS IIIC [5, 6, 7]), and N‐acetylglucosamine 6‐sulfatase (GNS; MPS IIID [8, 9]). Overall, the most common subtype is MPS IIIA, which has an incidence (or birth prevalence) of 0.68/100000 births in Australia, 0.8 in Taiwan and 0.1 in Japan ([10, 11, 12], respectively). In contrast, MPS IIIC is considerably rarer, with an incidence of 0.16 (Australia), 0.3 (Taiwan) and 0.04/100000 live births (Japan) ([10, 11, 12], respectively).

Whilst brain pathology has been reported in MPS IIIA in utero [13], patients with all four Sanfilippo subtypes appear apparently neurologically unaffected at birth. Those with rapidly progressing disease are commonly diagnosed around the age of 3–6 years (MPS IIIA; [14, 15]) or between 4 and 11 years (MPS IIIC; [16, 17]), although the age of symptom onset and the rate of disease progression can be highly variable in all MPS III subtypes. In early‐onset, rapidly progressing patients, first clinical signs include global developmental delay (particularly affecting speech) and behavioural difficulties including hyperactivity, aggression and neurocognitive impairment. Sleep disturbance is also notable, with motor function impaired later in the disease course.

Brain imaging studies, predominantly carried out in patients with MPS IIIA and MPS IIIB, indicate progressive loss of cerebral grey and white matter, reduced corpus callosum volume, and enlarged ventricles [18, 19, 20, 21]. Increased anterior and posterior pituitary volume was reported recently [22]. Interestingly, no abnormalities were reported in a single patient with MPS IIIC who underwent brain MRI [23]. The variability in disease presentation/progression in MPS IIIC patients, however, prevents the presumption that the findings in this case are representative of the subtype. More studies are needed. In the absence of approved treatments for brain disease, patients with MPS IIIA often die in their early/late teens [15, 24] or their 30s–40s (MPS IIIC [16]).

MPS III occurs naturally in animals, and a variety of laboratory animal model systems have also been created to enable the study of disease and evaluation of potential treatments. Species that demonstrate neurological impairment due to MPS III include flies ( *Drosophila melanogaster* ; [25, 26, 27]), zebrafish (*Danio. rerio*; [28, 29, 30]), mice [31, 32, 33, 34], birds (emu; *Dromaius. novaehollandiae*; [35]), goats [36], pigs [37], cows [38] and dogs [39, 40, 41].

The accumulation of partially degraded heparan sulfate is the hallmark of MPS III and leads to enlargement of the endo/lysosomal compartment, accompanied by secondarily stored gangliosides GM2 and GM3 for example, [31, 32, 42, 43], but not (as previously believed), unesterified cholesterol [44]. Intracellular dysfunction ensues, with impaired lysosomal function and endo/lysosomal trafficking [45], a block in autophagosome/lysosome fusion and impaired autophagic flux [43, 46], mitochondrial dysfunction [33, 47, 48] and aberrant cell–cell signalling [49]. Activation of inflammatory pathways [33, 47, 50, 51] occurs early in the brain disease process and recent post‐mortem evaluation of brain tissue has also demonstrated reduced myelination [52]. Gross cell death and brain atrophy is readily apparent in humans [18, 19, 20, 21] and larger model systems for example, MPS III dog brain [51], whereas reduced synaptic connections (dendritic spines) [49, 53, 54] appear to characterise murine brain disease.

At present there is no safe, effective treatment approved for any subtype of Sanfilippo syndrome/MPS III. Enzyme replacement, gene therapy, gene‐modified haematopoietic stem cell therapy and a variety of small molecules (e.g., genistein, trehalose, resveratrol) have been explored for their capacity to modify disease progression in animal model systems [55, 56, 57, 58, 59, 60, 61, 62, 63, 64, 65, 66, 67, 68, 69]. Human clinical trials of many of the approaches are ongoing.

Recent studies have indicated that small molecules or ‘nutraceuticals’ such as resveratrol, curcumin and trehalose can stimulate autophagy, improve energy metabolism, reduce oxidative stress and glial activation and as a result, modify disease progression and extend lifespan in neurodegenerative lysosomal storage disorder animal models including those with Batten disease, MPS VII and MPS III [64, 68, 70, 71, 72]. Supporting these observations, data emerging from small human clinical trials indicate that trehalose may also be beneficial in patients with Niemann‐Pick disease and MPS III [73, 74] although larger, randomised placebo‐controlled, double‐blind trials are needed to verify these early findings.

We were therefore interested to observe that dietary supplementation with polyamines such as spermidine (3 mM in drinking water) enhanced autophagic flux and mitophagy in aged C57Bl/6 mouse cells, improving mitochondrial volume and oxygen consumption, and reducing inflammation (as indicated by plasma TNFα levels), extending median lifespan when provided either for life or only later in life [75]. In cell and animal models of Alzheimer's disease, spermidine supplementation reduced NF‐_Κ_B‐mediated inflammasome assembly and lowered pro‐inflammatory cytokine expression [76], and increased autophagic flux and reduced oxidative stress and lipid peroxidation in a concentration‐dependent manner [77]. Further, in a rotenone‐induced rat model of Parkinson's disease [78], spermidine‐treated animals displayed improved motor function and reduced oxidative stress markers along with lower pro‐inflammatory cytokine expression. Again, the findings were dose dependent. A diet rich in spermidine has also been found to be associated with increased longevity [79].

Here, we showed that whilst spermidine levels were not different in diseased versus non‐diseased flies, all animals exhibited significantly reduced spermidine levels with age. Thus, we supplemented MPS III flies or mice with oral spermidine and subsequently examined the short‐term impact on function (MPS IIIA and MPS IIIC flies) and brain pathology (MPS IIIA mice). Whilst further longer‐term dose‐finding studies and evaluation of the impact of spermidine treatment on clinical disease in mice with MPS III are required, our preliminary data indicate that spermidine supplementation inhibits astrogliosis and may slow disease progression in this presently untreatable disorder.

## 2 Materials and Methods

### 2.1 Approvals

This research protocol was approved by the Institutional Animal Ethics and Biosafety Committees prior to study commencement.

### 2.2 Drosophila Stocks

Previously characterised *D. melanogaster* models of MPS IIIA [25] and MPS IIIC [26] were maintained at 18°C in 60% humidity on a 12‐h light/dark cycle. Flies were turned into fresh vials containing fortified medium (1% agar, 1% glucose, 6% fresh yeast, 9.3% molasses, 8.4% course semolina, 0.9% acid mix and 1.7% tegosept) every 3–4 days.

### 2.3 Metabolomics

Whole flies aged 0, 8, 15 and 42 days (*n* = 5 biological replicates, each containing *n* = 3 flies/genotype and age) were provided to Metabolomics Australia for sample processing and metabolite extraction. Briefly, flies were added to pre‐chilled cryomill tubes (temperature < −10°C) containing 600 μL of extraction solution (methanol: water, 3:1 v/v, containing internal standard mixture of 2 μM 13C5, 15 N1 Valine, 2 μM of 13C6‐Leucine and 3 μM of 13C6‐Sorbitol). The samples were then homogenised at 6800 rpm using three 30 s pulses with 45 s intervals between pulses. The resulting homogenate (480 μL) was transferred to a fresh microfuge tube on ice. Then, 120 μL of chloroform was added (chloroform:methanol:water ratio of 1:3:1 (v/v) monophasic mixture) and the samples were vortexed vigorously. The mixture was incubated on ice for 10 min with intermittent mixing, then centrifuged at maximum speed for 5 min at 0°C. The supernatant was collected into fresh Eppendorf tubes held on ice. At this stage, 100 μL aliquots were taken from each supernatant sample and combined to create a pooled biological quality control (PBQC). For drying, 400 μL of the remaining supernatant was transferred sequentially in 50 μL aliquots into a pulled point insert. Samples were dried in a speed vacuum at 35°C, with additional 50 μL aliquots added every 30–40 min until the total volume was completely dry. A final preparatory drying step was executed by adding 50 μL of methanol and evaporating to dryness to ensure samples were moisture‐free prior to derivatisation with methoxyamine and BSTFA +1% TMS. The derivatised samples were analysed using a GC‐QQQ 8050 MS (Shimadzu). The sample analysis order was randomised to minimise analytical bias. To monitor instrument stability and performance, a PBQC sample was analysed every six experimental samples.

#### 2.3.1 Data Preprocessing and Quality Control

Technical reproducibility of the metabolite intensity data was verified by assessing the stability of internal standards and PBQC samples. All QC features demonstrated a coefficient of variation (CV) < 20%. Prior to statistical analysis, the metabolomics dataset underwent median normalisation, log10 transformation and autoscaling (unit variance scaling) to account for heteroscedasticity and differences in metabolic abundance scales. Unsupervised multivariate analysis was performed via principal component analysis (PCA) at each age (Days 0, 8, 15 and 42) to assess sample clustering and identify potential outliers.

#### 2.3.2 Differential Abundance Analysis

Differential abundance of metabolites was determined using moderated *t*‐tests implemented in *limma* [1]. An intercept‐free design matrix was constructed to model group‐specific effects, and a contrast matrix was implemented to calculate the differences between each MPS III model (MPS IIIA and MPS IIIC) and its respective age‐matched control at each time point (D0, D08, D15 and D42). Linear models were fitted for each metabolite, and empirical Bayes moderation was applied with robust estimation to shrink the metabolite‐wise variances towards a common value, improving stability for small sample sizes [80]. Statistical significance was defined using a False Discovery Rate (FDR) threshold of < 0.05 after Benjamini‐Hochberg adjustment [81]. Additionally, a targeted analysis was performed for the polyamines spermidine and putrescine. The normalised abundances for each of these metabolites were subjected to two‐way ANOVA and pairwise post hoc comparisons between genotypes at each age were performed using independent *t*‐tests with Bonferroni adjustment for multiple testing. *p* < 0.05 was regarded as statistically significant.

#### 2.3.3 Pathway Enrichment Analysis

Pathway analysis was conducted using *limma::fry* [82] to perform self‐contained metabolite‐set testing against the KEGG pathway database [83]. To ensure robust biological inference, only KEGG pathways containing at least three metabolites detected within the *Drosophila* dataset were included in the analysis. A directional hypothesis was applied to identify pathways significantly shifted in either direction. KEGG pathway metabolite sets were considered significantly altered if they achieved an FDR‐adjusted *p*‐value of < 0.05.

### 2.4 Drug Treatment—Drosophila

**FIGURE 1:**
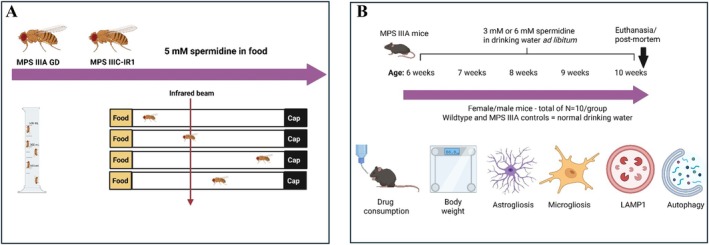
Overview of the MPS IIIA and MPS IIIC fly (A), and MPS IIIA mouse spermidine supplementation experiments (B). MPS IIIA GD and MPS IIIC‐IR1 flies were treated with 5 mM spermidine in food. The MPS IIIA and MPS IIIC drosophila models have been characterised previously [25, 26]. MPS IIIA mice are pre‐symptomatic to 12 weeks of age. Treatment was provided from 6 to 10 weeks of age, via supplementation in drinking water. Consumption was monitored via weighing of water bottles; body weight was evaluated, and at post‐mortem, tissue pathology was examined. Figures generated in Biorender.com.

For drug treatments, flies were laid and reared in 5 mM spermidine‐supplemented food (3% agar, 6% sucrose), with spermidine sourced from Sigma (#S2626). Controls were raised on the same (non‐supplemented) food. An overview of the spermidine supplementation experiments in flies is shown in Figure 1.

### 2.5 Climbing Assays

Climbing assays were performed as described previously [25]. The total number of flies per genotype was *n* = 30–60. Sets of 11–16 flies were transferred into a glass 500 mL measuring cylinder and after a three‐minute recovery period, the flies were tapped to the bottom of the cylinder. The proportion of flies that climbed above 300 mL was determined at 10 s intervals for a total of 90 s. Male Elav2 > MPS IIIA flies (and controls) were tested at 15 days. Male Elav2 > MPS IIIC flies (and appropriate controls) were tested at 8 days.

### 2.6 Drosophila Activity Monitor Test

Using a previously published method [26], individual flies (0–3 day old) were placed in glass tubes containing food with or without spermidine (5 mM), housed in a Trikinetics *Drosophila* Activity Monitor (DAM) system (Trikinetics, MA, USA). Activity was monitored at 25°C with a 12‐h light/dark cycle for 7 days. Movement of the flies interrupted an infrared beam that bisected the glass tubes enabling average activity to be determined. FaasX (Paris‐Saclay Institute of Neuroscience, Saint‐Aubin, France) was used to examine circadian rhythms and locomotor activity, calculated in 30‐min epochs. The average activity during the lights on (day) and lights off (night) period was also determined for flies with each genotype.

### 2.7 Mouse Breeding, Husbandry and Drug Treatment

An overview of the mouse experiments is shown in Figure 1. The mice were sourced from a breeding colony of congenic MPS IIIA mice (B6.Cg‐Sgsh^mps3a^) maintained in the Flinders University College of Medicine and Public Health Animal Facility, South Australia. Mice were group‐housed in a temperature/humidity‐controlled room on a 14‐h light: 10‐h dark cycle, with food and water available *ad libitum*. Mice received toilet rolls and red transparent plastic houses, plus nesting material for environmental enrichment. Genotyping for the *SGSH* mutation was undertaken on ear‐notch tissue [84]. All breeding, housing and experimental procedures complied with the Australian code for the care and use of animals for scientific purposes (8th edition; 2013; updated 2021).

Cohorts of six‐week‐old male/female MPS IIIA mice were placed on spermidine‐supplemented drinking water (either 3 or 6 mM) provided in brown glass bottles to protect the contents from light exposure. The spermidine was sourced from Cayman Chemical (#14918) and aliquots of concentrated stock solution were stored at −80°C until they were thawed and diluted for use. MPS IIIA mice aged 6–10 weeks have been shown to be at an early stage in the murine disorder [51]. In these mice, significant amounts of substrate (heparan sulfate) accumulate in the brain, and the neuroinflammatory response (micro‐ and astro‐gliosis) is robust, but overt clinical signs are not yet apparent. Selection of mice at this early disease stage enabled examination of the capacity of a treatment to reverse or slow the appearance of disease lesions. Control animals (unaffected and MPS IIIA of both genders) received unmodified drinking water. The drugged water was made up and changed twice a week, and drug delivery continued from 6 to 10 weeks of age. At this point, humane euthanasia was performed (using CO_2_ asphyxiation) to enable post‐mortem tissue sampling and an evaluation of the impact of treatment on disease lesions in brain.

### 2.8 Post‐Mortem Tissue Sampling

At post‐mortem, following cardiac perfusion with ice‐cold phosphate buffered saline, the brain was removed and cut down the midline. The right hemisphere was immersion‐fixed in 4% paraformaldehyde for 48 h at 4°C, prior to processing into paraffin. The left hemisphere was placed in a mouse brain blocker, and five 2‐mm hemi‐coronal sections were made and frozen for subsequent biochemical evaluation.

### 2.9 Immunohistochemistry and Histochemistry

Previously published methods were used to visualise MPS IIIA‐related disease lesions in brain [45, 85]. Six micron‐thick sections of brain were cut on a rotary microtome (Thermo Scientific Microm HM325 rotary microtome, Wetzlar, Germany) and mounted on glass slides (Superfrost Plus, Thermo Scientific, USA). Brain sections were stained with glial fibrillary acidic protein (GFAP) or lysosome‐associated membrane protein‐1 (LAMP1) antibodies and isolectin B4. Biotinylated species‐specific secondary antibodies, 1:2000 (Jackson ImmunoResearch Labs), Vectastain Elite ABC kit reagents (PK‐6100; Vector Laboratories, CA, USA), and the diaminobenzidine (DAB) liquid substrate chromagen system (#3468; Dako, Glostrup Denmark) were used to amplify the reaction and provide chromogenic visualisation.

Staining was batched, with analysis carried out by a researcher blind to mouse genotype, gender and treatment status. Sections were viewed on an Olympus BH2 microscope, with images captured using an Olympus DP22 camera and Olympus CellSens imaging software. The number of isolectin B4‐stained cells per unit area of brain was counted manually. Threshold analysis of GFAP and LAMP1 staining was performed based on the optical density of positive immunostaining, reported as % immunoreactivity, using FIJI software.

### 2.10 Western Blots

Mouse cerebella were thawed on ice and homogenised using a handheld motorised pestle in 1 mL of RIPA buffer (89900, Thermo Scientific, USA) supplemented with 1× cOmplete Protease Inhibitor Cocktail (11697498001, Roche, Switzerland). The lysates were centrifuged at 15000 × g for 5 min at 4°C to pellet insoluble material. Cleared lysates were sonicated using a Bioruptor (UCD‐200, Diagenode, USA) for 5 min (30 s on, 30 s off) in an ice‐water bath, followed by centrifugation at 16000 × g for 5 min at 4°C. Total protein concentration was estimated using the EZQ Protein Quantitation Kit (R33200, Thermo Fisher).

Samples (60 μg total protein per cerebellum sample) were prepared in a final volume of 30 μL containing 1× lithium dodecyl sulfate (LDS) sample buffer (NP0008, Thermo Fisher, USA) and Sample Reducing Agent (NP0009, Thermo Fisher, USA). The samples were heated at 95°C for 5 min, centrifuged at 15000 × g for 5 min at room temperature, and loaded into the wells of pre‐cast 12% NuPAGE Bis‐Tris gels (NP0342, Thermo Fisher, USA) along with 3 μL of Colour Prestained Protein Standard (P7719, New England Biolabs, USA). Protein separation was carried out in a Bolt Mini Gel Tank at 120 V for 2 h in ice‐cold NuPAGE 1× MOPS SDS Running Buffer (NP0001, Thermo Fisher, USA) supplemented with NuPAGE Antioxidant (NP0005, Thermo Fisher, USA). Proteins were transferred to PVDF membranes (0.2 μm, 162‐0177, Bio‐Rad, USA) using ice‐cold 1× Tris‐Glycine SDS buffer containing 20% methanol (Supelco, USA) at 20 V for 1 h at 4°C.

The blots were blocked with 5% skim milk powder in 1× tris‐buffered saline with 1% Tween 20 (1× TBST) for 1 h at room temperature with gentle agitation. Following blocking, they were incubated overnight at 4°C in primary antibody solutions (Rabbit anti‐LC3A; Novus NB100‐2331; 1/500 or Rabbit anti‐SQSTM1/p62; CST, 5114; 1/1000 or Mouse anti‐Tubulin; DSHB, E7; 1/2000) prepared in 5% skim milk powder in 1× TBST, with gentle agitation. Subsequently, the blots were incubated in secondary antibody solutions (Goat anti‐rabbit IgG peroxidase; Sigma A0545; 1/50000 or Donkey Anti‐mouse IgG; Rockland, 610‐703‐124; 1/10000), also prepared in 5% skim milk powder in 1× TBST. Between each blocking and antibody incubation step, the blots were rinsed three times and washed four times for 10 min each in 1× TBST at room temperature with gentle agitation. Peroxidase signals were detected using Pierce ECL substrate (32106, Thermo Fisher, USA) and visualised with a ChemiDoc Imaging System (Bio‐Rad, USA). The background‐subtracted densities of each band were measured in ImageLab (Bio‐Rad, USA).

### 2.11 Statistics

Data were analysed using parametric statistical tests (*t*‐tests when comparing between two groups or ANOVA with post hoc Bonferroni for multiple comparisons). GraphPad Prism Software and Microsoft Excel were used. *p* < 0.05 was considered statistically significant.

## 3 Results

### 3.1 Metabolomic Analysis in Whole MPS III Flies

**FIGURE 2:**
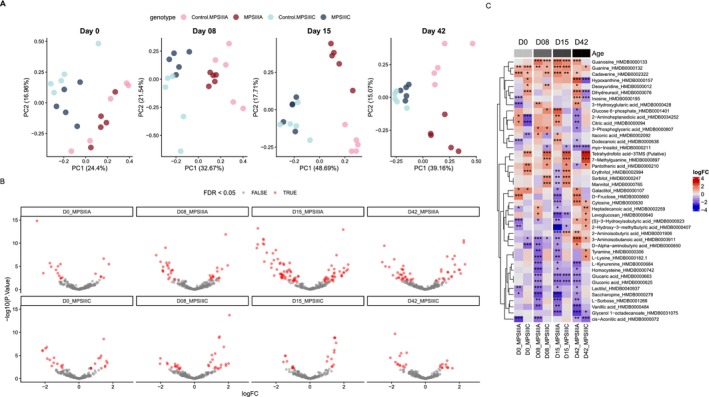
Multivariate and univariate analysis of the *Drosophila* MPS III metabolomes. (A) Principal Component Analysis (PCA) biplot of normalised *Drosophila* metabolomes at each developmental stage. Individual points represent biological replicates, colour‐coded by genotype. Percentages of total variance explained by Principal Component 1 (PC1) and PC2 are displayed on the respective axes. (B) Volcano plot illustrating differential metabolite abundance between MPS III *Drosophila* and age‐matched controls. The y‐axis displays statistical significance ‐log10 (*p*‐value), and the x‐axis displays the magnitude of change log2 fold change (logFC). Points are colour‐coded based on a False Discovery Rate (FDR) adjusted *p*‐value threshold of < 0.05. Differential expression was determined using moderated *t*‐tests (limma) with FDR correction for multiple comparisons. (C) Heatmap of differentially abundant metabolites. Inclusion was restricted to metabolites significantly altered in at least 3 genotype‐control comparisons. Cell colour represents the logFC, statistical significance is indicated by asterisks: *p < 0.05, **p < 0.01, ***p < 0.001.

**FIGURE 3:**
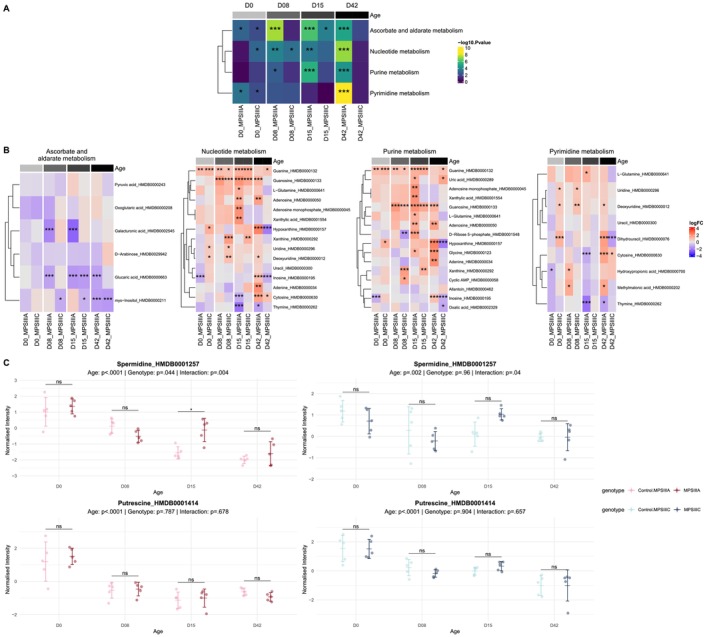
Pathway enrichment and polyamine metabolism in MPS III Drosophila. (A) Heatmap of significantly enriched KEGG pathways. Pathways shown were significantly altered in at least 3 MPS III Drosophila comparisons. Cells are colour‐coded and labelled by statistical significance. (B) Heatmaps displaying the log2 fold change (logFC) of individual metabolites within the significantly enriched KEGG pathways identified in (A). Significance levels are denoted as: *p < 0.05, **p < 0.01, ***p < 0.001. (C) Relative abundance of spermidine and putrescine in MPS III *Drosophila* models compared to controls across the lifespan. Global effects of age, genotype, and their interaction (Age × Genotype) were determined via two‐way ANOVA and are indicated in the panel headers. Pairwise post hoc comparisons between genotypes at each age were performed using independent *t*‐tests with Bonferroni adjustment for multiple testing (*p < 0.05; ns, not significant). Data are presented as mean ± standard deviation.

Evaluation of the metabolome of MPS IIIA and MPS IIIC flies demonstrated that there are significant aberrations in metabolite expression in diseased versus control flies, and that the metabolic derangement occurs early that is, is present at day 0 (Figures 2 and 3). The alteration in metabolite levels was not consistent between MPS IIIA and MPS IIIC flies, as indicated by the principal component analysis (Figure 2A), and MPS IIIA flies appeared to exhibit greater downregulation of metabolite expression, than was observed in MPS IIIC flies (Figure 2B,C). Conversely, other than at 42 days, the MPS IIIC flies tended to exhibit greater up‐regulation of metabolite expression compared with MPS IIIA flies (Figure 2C). The full outputs of differential metabolite expression analyses can be found in File S1.

Overall, the most significantly up‐regulated metabolites were those involved in nucleotide and purine metabolism (guanine and guanosine) (Figure 3B), and the most significantly down‐regulated metabolites (particularly in MPS IIIA flies) were those involved in ascorbate and aldarate metabolism, including glucaric acid and myo‐inositol (Figure 3A,B). The full output of pathway analysis of the metabolomics data can be found in File S2.

Whilst differential expression of the metabolites spermidine and putrescine was not consistently observed in MPS IIIA or MPS IIIC flies c.f., control flies, we noted a highly significant reduction in the level of both polyamines with age in all animals (Figure 3C). Spermine was not measured in the metabolomic study.

### 3.2 Effect of Oral Spermidine Treatment on Fly Behaviour

**FIGURE 4:**
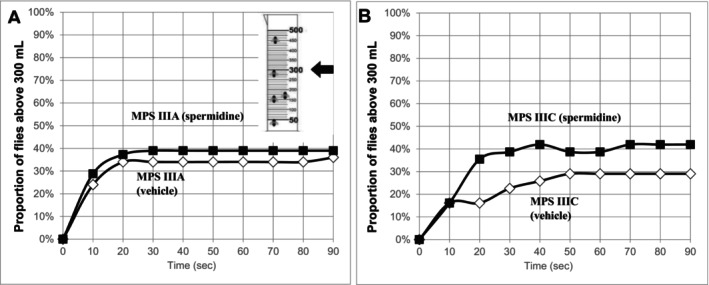
Effect of oral spermidine on climbing ability in MPS IIIA GD (A) and MPS IIIC‐IR1 (B) flies. Spermidine (5 mM) was supplied in food throughout the study period. Flies were added to the measuring cylinder and tapped to the bottom. The proportion of flies that climbed above the 300 mL mark was quantified at 10 s intervals up to 90 s. Spermidine treatment appeared to slightly improve the ability of flies to climb, particularly those with MPS IIIC. Flies were aged 15‐ or 8‐days (MPS IIIA, MPS IIIC, respectively) at the time of testing.

MPS IIIA flies have previously been shown to have climbing defects that become apparent at day 1 but become more pronounced with age [25]. As shown in Figure 4A, spermidine treatment of MPS IIIA flies led to a small (~5%) increase in the proportion of Day 15 flies that were able to climb above the 300 mL mark in the measuring cylinder. The effect of drug treatment was more pronounced in the Day 8 MPS IIIC flies (Figure 4B) with ~40% of the treated MPS IIIC flies able to successfully climb (versus 30% of the untreated MPS IIIC controls).

**FIGURE 5:**
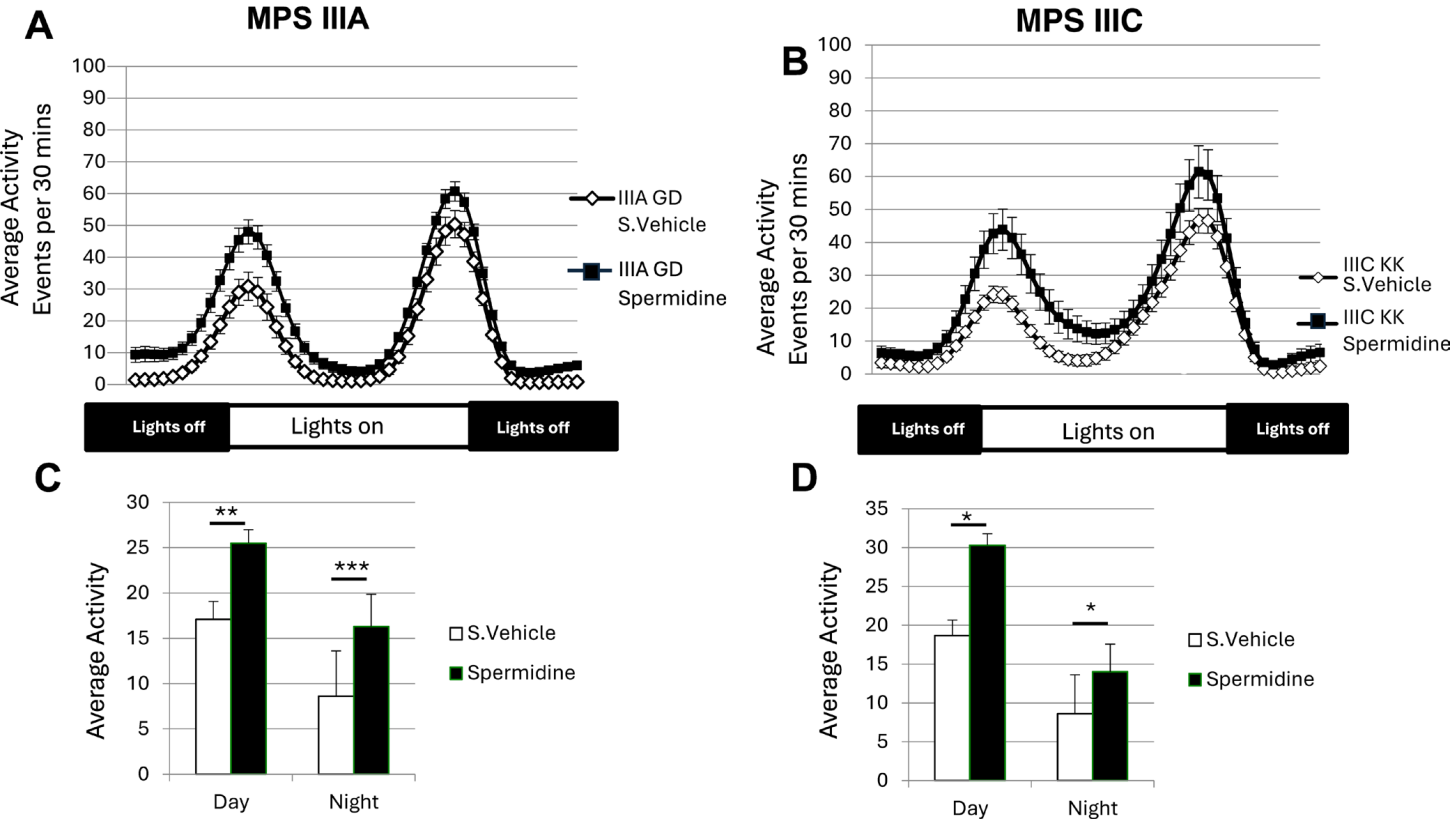
Effect of oral spermidine (5 mM) on the overall activity of MPS IIIA GD (A) and MPS IIIC‐IR1 (B) flies, measured in the drosophila activity monitor. Flies with both MPS IIIA and MPS IIIC have previously been found to be hypoactive relative to the unaffected controls. Activity in the day (lights on) and night (lights off) epochs is shown in C and D (for MPS IIIA and MPS IIIC flies respectively). Spermidine treatment significantly increased the activity of MPS IIIA and MPS IIIC flies in the day (lights on) and night (lights off). Data is mean ± SD. *p < 0.05, **p < 0.01, ***p < 0.001.

*Drosophila* activity monitor studies have shown that both MPS IIIA and MPS IIIC flies exhibit reduced overall activity [26, 86]. There was a statistically significant improvement in Drosophila activity following spermidine treatment (Figure 5). In the plots of 24‐h average activity, MPS IIIA and MPS IIIC flies showed increased overall activity (Figure 5A,B, respectively), with the impact of the treatment more pronounced in the MPS IIIC flies. When the lights on and lights off data was summed for each phase, spermidine treatment significantly increased the activity of both MPS IIIA and MPS IIIC flies (Figure 5C,D, respectively), in both phases, but the improvement was most significant during the lights‐on phase.

### 3.3 Palatability and Short‐Term Safety of Oral Spermidine in MPS IIIA Mice

**FIGURE 6:**
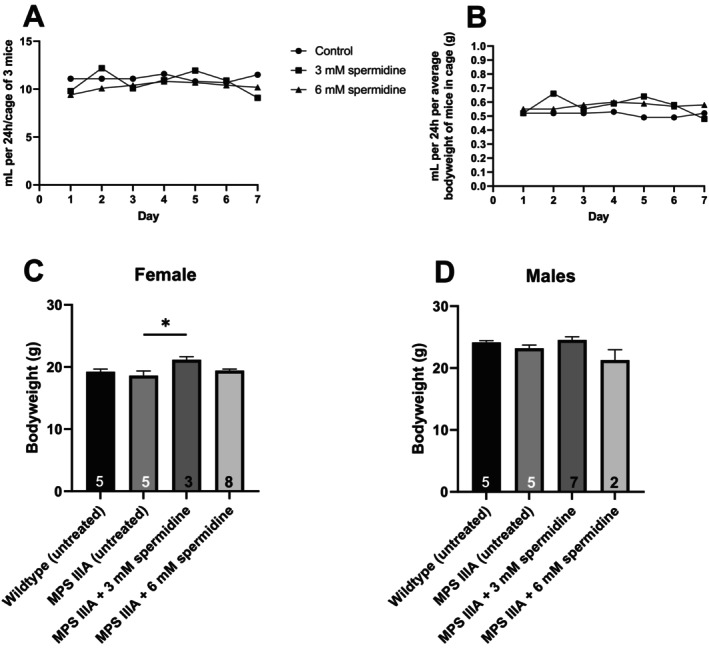
The consumption of spermidine (3 or 6 mM) in mouse drinking water provided in brown glass bottles, was determined to ensure consumption (A; mL per 24 h; cage of 3 mice). The consumption corrected for mouse bodyweight is shown in B. Drugged water was changed every 2–3 days. A comparison of bottle weight was made with age‐matched mice receiving normal drinking water (control/untreated). *N* = 3 mice/cage. The effect of oral spermidine treatment on female (C) and male (D) MPS IIIA mouse bodyweight (g) was also determined. Data are mean ± SD. The number of mice per group is shown in each bar. Only significant comparisons are shown. *p < 0.05.

To explore the efficacy of oral spermidine further, we provided MPS IIIA mice of both genders with one of two doses of spermidine in their drinking water (3 or 6 mM). A pilot study was first carried out in *n* = 3 mice/group (MPS IIIA control, MPS IIIA 3 mM and MPS IIIA 6 mM) to ensure the palatability of the spermidine in the drinking water. Water bottles were weighed daily for a week to ensure drinking occurred in spermidine‐supplemented mice in a way that was compatible to control water‐fed mice (Figure 6A). No reduction in drinking of either dose of spermidine‐supplemented water was observed, either on a mL/cage basis or a mL/bodyweight basis (Figure 6B). Similarly, when the bodyweight of the mice was evaluated over the full course of the four‐week study (*n* = 10 mice/group; Figure 6C,D), no reduction in bodyweight due to failure to drink or ill‐health due to treatment was noted in mice of both genders, and all mice in the study remained healthy throughout the four‐week treatment period; therefore, we concluded that these doses of spermidine when provided in drinking water are safe in the short‐term at least. Others have used these same doses to treat 18‐month‐old C57Bl/6 mice for 6‐months, without incident [87].

### 3.4 Effect of Short‐Term Oral Spermidine on MPS IIIA Mouse Brain

**FIGURE 7:**
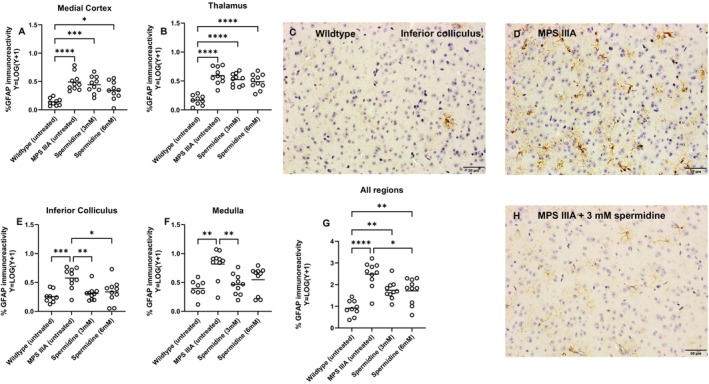
Effect of oral spermidine (3 or 6 mM) on GFAP expression in MPS IIIA mouse brain. The staining was examined by an experimenter blind to genotype/treatment group. (A) medial cerebral cortex, (B) thalamus, (E) inferior colliculus, (F) medulla and (G) all areas combined. *p < 0.05, **p < 0.01, ***p < 0.001, ****p < 0.0001. Only statistically significant comparisons are shown. Each dot is a mouse. Bar = mean. Representative images of the GFAP staining in inferior colliculus are shown in C, D, H.

Short‐term oral spermidine treatment of MPS IIIA mice led to a statistically significant reduction in GFAP expression regionally within treated mouse brain (Figure 7A–H). Whilst no significant reduction was observed in the cerebral cortex and thalamus, staining intensity was trending downwards with increasing concentrations of spermidine (Figure 7A,B). On the other hand, statistically significant reductions in astrogliosis were apparent in the inferior colliculus and the medulla of treated MPS IIIA mice when staining was compared with that seen in the control MPS IIIA animals (Figure 7C–E,H). There was no impact of dose seen in the treated inferior colliculus and medulla, with both 3‐ and 6‐mM spermidine resulting in similar outcomes.

**FIGURE 8:**
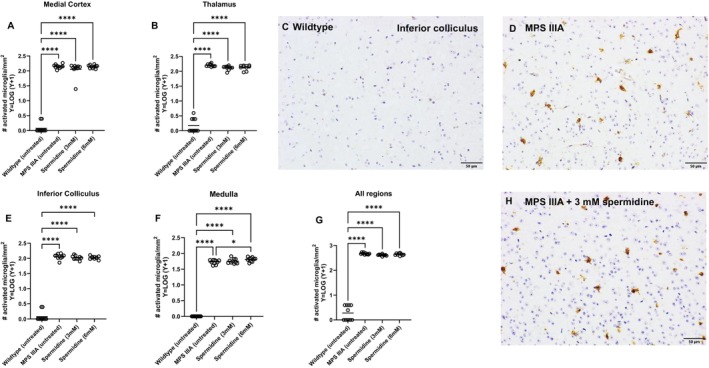
Effect of oral spermidine (3 or 6 mM) on the number of isolectin‐B4‐stained (activated) microglia in MPS IIIA mouse brain. The staining was examined by an experimenter blind to genotype/treatment group. (A) medial cerebral cortex, (B) thalamus, (E) inferior colliculus, (F) medulla and (G) all areas combined. Each dot is a mouse. Bar = group mean. Representative images of the isolectin B4 staining in inferior colliculus are shown in C, D, H. Only statistically significant comparisons are shown. *p < 0.05, ****p < 0.0001.

**FIGURE 9:**
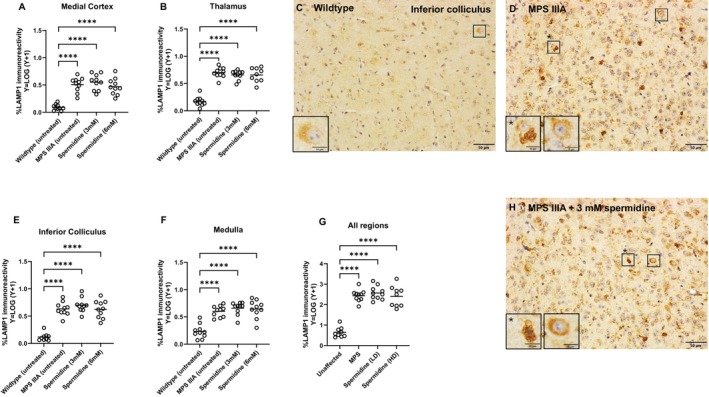
Effect of oral spermidine (3 or 6 mM) on LAMP1 expression in MPS IIIA mouse brain. Staining was examined by an experimenter blind to genotype/treatment group. (A) medial cerebral cortex, (B) thalamus, (E) inferior colliculus, (F) medulla, and (G) all areas combined. *p < 0.05, ****p < 0.0001. Each dot is a mouse. Bar = group mean. Representative images of the LAMP1 staining in inferior colliculus are shown in C, D, H.

In contrast, short‐term spermidine had no impact on microgliosis in the MPS IIIA brain (Figure 8) with isolectin B4‐stained microglia remaining morphologically ‘activated’ that is, enlarged cell body, shortened cell processes (Figure 8C,D,H), present in similar numbers in MPS IIIA mice treated with both doses of spermidine, and untreated MPS IIIA mice. The number of activated microglia in the brain of both MPS IIIA mouse groups was highly significantly elevated in comparison to the control unaffected group (Figure 8A,B,E–G). Similarly, when we evaluated the enlarged lysosomal compartment that is a hallmark of MPS IIIA and other similar disorders with LAMP1 staining (Figure 9), we found there was no reduction in compartment size in spermidine‐treated MPS IIIA mice compared to control MPS IIIA mice. Both groups exhibited highly significant expansion of the lysosomal compartment compared to unaffected control mice as shown in the representative images (Figure 9C,D,H).

Finally, when we examined the impact of short‐term oral spermidine on LC3II/I and p62 levels in a small cohort of mouse cerebella samples (*n* = 3) via western blot (Figure S1), whilst LC3‐II/LC3‐I ratios were elevated in 10‐week‐old MPS IIIA mouse brain by ~four‐fold c.f. unaffected controls (A), the levels failed to reach statistical significance. Oral spermidine treatment (3 mM but not 6 mM) further elevated the LC3‐II/LC3‐I ratio (*p < 0.05) although a large amount of variability was observed between the samples, lowering confidence in the outcome. The level of the autophagy substrate p62 was unchanged in the 10‐week‐old untreated MPS IIIA mouse cerebellum c.f. unaffected control tissues (B), and there was no difference in the MPS IIIA mouse p62 expression in cerebellum samples post‐treatment.

## 4 Discussion

To the best of our knowledge, this is the first metabolomic evaluation performed on a *Drosophila* model of a mucopolysaccharide‐storing disorder. The findings indicate significant aberration in the MPS IIIA and MPS IIIC whole fly metabolome, evident from Day 0. A statistically significant reduction in the level of metabolites involved in the ascorbate and aldarate pathways was noted, and altered nucleotide, purine and pyrimidine metabolism was also seen.

Myo‐inositol deficiency may conceivably be due to impaired biosynthesis from glucose, reduced uptake in the gut and/or increased elimination/excretion by the kidneys. Presumptively, the deficiency in glucaric acid is linked to that of myo‐inositol, which is a precursor. Significantly reduced myo‐inositol levels have also been reported by Fu and colleagues in evaluations of MPS IIIA and MPS IIIB patient [88], and MPS IIIB mouse sera samples [89]. In the latter study, AAV‐based NAGLU gene replacement ameliorated the myo‐inositol (and neurological) deficits. Adult patients with galactosemia who have been treated with a galactose‐restricted diet exhibit reduced white matter, cerebellar and putamen myo‐inositol levels, and it has been hypothesised that this deficiency underlies the concurrent cerebral white matter and sub‐cortical grey matter loss [90]. Supplementary feeding of myo‐inositol to mice with galactosemia led to improvements in brain structure, anxiety and motor function [91, 92]. The impact of myo‐inositol deficiency in animal models and patients with MPS III requires further exploration, as it may represent a safe, effective way to at the very least, slow disease progression.

The elevation of various purine metabolites (principally guanine and guanosine, but also adenosine, xanthine, adenine, adenosine monophosphate and uric acid) may be a (neuro)protective effort by organisms aimed at reducing oxidative stress [93, 94], which has been described in MPS III (e.g., 47). The outcomes require further evaluation however, as they are at odds with previously published serum metabolomic data obtained from MPS IIIA patients and MPS IIIB patients and mice [88, 89]. In those studies, purine (and pyrimidine) metabolites were largely reduced in MPS III sera samples, compared to control levels. Evaluation of the metabolome in MPS III animal model brain tissue would help to clarify the scope and nature of the involvement of these pathways in MPS III pathogenesis.

In a preliminary study, we have also examined the impact of short‐term oral spermidine supplementation on disease presentation in three pre‐clinical models of Sanfilippo syndrome (MPS IIIA and MPS IIIC flies and MPS IIIA mice). Our exploratory study sought to determine whether, as has been reported for other dementia‐causing disorders, spermidine supplementation could prevent or slow the appearance of brain disease markers. Should that be the case, it would warrant longer‐term, larger‐scale evaluation of the capacity of this polyamine to ameliorate the clinical signs resulting from neurodegenerative changes.

Whilst spermidine levels were not consistently significantly different in whole flies with MPS IIIA or MPS IIIC c.f. unaffected controls at any age up to 42 days (Figures 2 and 3), we did note a significant reduction in spermidine (and putrescine) concentration with age in all fly lines (regardless of genotype). The latter observation agrees with previous findings in normal fly heads [95] and unaffected rat brain [96]. No significant reduction in spermidine concentration was noted in human occipital cortex from adulthood to old age [97], however significant reductions in spermidine levels have been noted in human basal ganglia [98]. Thus, supplementation of spermidine in our experiments was not rectifying a disease‐related deficit, rather, it sought to enhance the level of a polyamine that decreases in concentration with normal ageing.

Improvements in neurological function (climbing behaviour and motor activity) were observed in short‐term spermidine‐treated *Drosophila* models of MPS IIIA and MPS IIIC and reduced astrogliosis was observed in the brain of spermidine‐treated MPS IIIA mice. We note, however, that there was no reduction in lysosomal compartment size nor any disease ameliorating effect on microglial activation or LC3‐II/LC3‐I ratios in MPS IIIA mouse brain after 4 weeks of treatment. A longer treatment duration and/or a different dose may assist; dose‐dependence has been reported in previous Alzheimer disease model studies of spermidine [76, 77] and the mouse brain concentration of orally supplied deuterated spermidine was observed to increase with treatment duration, with higher d8‐spermidine concentrations seen after 8‐weeks versus 1‐ or 4‐weeks of treatment [87]. Further experiments are required to more fully characterise the response to spermidine treatment in MPS III model systems and dose‐ranging clinical‐behavioural studies carried out over longer periods now appear warranted given these preliminary findings, and the apparent amenability of spermidine supplementation in humans. MPS III is untreatable at present; therefore, any cheap, widely available, and safe intervention that slows disease progression is highly desirable.

Only limited studies have been performed in humans to evaluate the impact of spermidine supplementation on cognition or dementia‐related pathologies and there is only one report of spermidine treatment in children or adolescents [99] to the best of our knowledge. In a year‐long double‐blind placebo‐controlled trial in older adults with subjective cognitive impairment [100], spermidine treatment (0.9 mg/day) was found to be safe but was unable to improve mnemonic performance. The authors suggest that there was potentially an improvement in inflammation and verbal memory; however, higher dose studies are needed. In a more recent unblinded study [101], older adults receiving a higher dose of spermidine (3.3 mg/day) displayed a significant improvement in the mini mental state examination after 1 year of treatment when data were compared to baseline test results. Finally, a girl (aged 6.7 years) and an adolescent (aged 16.2 years) with GRIN2B loss‐of‐function mutations that resulted in a developmental disorder characterised by the presence of autistic traits, impulsivity, mild hyperactivity and attention deficits received 3 mg/day spermidine provided in food for a total of 18 months [99]. The treatment was found to be safe and resulted in a within‐subjects improvement in the standard scores of the adaptive behaviour composite of the Vineland Adaptive Behaviour Scale (VABS‐II). Increased scores in the communication and daily living domains were responsible for the standard score improvement. Supporting this, the study participant's parents indicated that there were improvements in social communicative interactions and daily domestic autonomy.

The latter study raises an intriguing possibility, that spermidine's actions in the MPS IIIA and MPS IIIC flies may be due to modification of (presumptively aberrant) glutamate signalling. Spermidine has been shown to positively modulate NMDA receptor activation, increasing calcium influx [102]. In one MPS IIIB mouse study, brain levels of glutamate were reported to be elevated [103], but serum glutamate levels have also been found to be significantly lower in MPS IIIA and MPS IIIB patients and MPS IIIB mice, compared with controls [88, 89]. In our metabolomic evaluation of MPS IIIA and IIIC flies (Figure 3C), we found that levels of the glutamate precursor glutamine were significantly increased in MPS IIIA flies, but only at one of the four ages evaluated (Day 15). The fact that whole fly preparations were evaluated here likely precludes firm interpretation of the outcomes with respect to brain glutamate, and examination of fly heads should be performed in future analyses.

With respect to glutamate receptors, AMPA (but not NMDA) receptor subunit levels in MPS IIIA models have been evaluated both in vitro and ex vivo [94]. Elevated GluA2 (but not GluA1) receptors on glial cell membranes were observed in MPS IIIA iPSC‐derived neural cultures; however, there was no change in the amount or distribution of receptors in murine MPS IIIA cerebral cortex. On the other hand, the concentration of both GluA1 and GluA2 subunits of AMPA receptors and the NR1 subunit of NMDA receptor progressively decreases in the hippocampus of a mouse model of MPS VII (from 8 to 28 weeks of age) [104]. As MPS VII is a similar disorder to MPS IIIA, it is tempting to speculate that similar outcomes might be seen should the same experiments be repeated on MPS IIIA mouse hippocampus. Examination of hippocampal tissue subjected to spermidine treatment would enable us to understand whether NMDA receptor stimulation contributes to the clinical impact of the polyamine in seen in MPS III flies.

Spermidine can be synthesised endogenously within cells from the amino acids arginine, ornithine and methionine [105] and microbial synthesis also occurs in gut. Spermidine is also available in foodstuffs (reviewed in [106]), with vegetable sprouts, peas, mushrooms, broccoli, wheatgerm and soybeans providing high quantities per gram of food although wholegrain bread, apples, pears, potatoes and salads provide the main source of spermidine in the diet (when % of intake is considered). Daily intake is variable based on regional diets but appears to be of the order of ~5–26 mg/day (reviewed in [106]). Serum levels of spermidine have been reported to reduce with age [107].

Whilst spermidine levels in serum were found to correlate with mini‐mental state exam score in a study of 80 older adults (aged 60–96 years [107]), in another study of 659 subjects aged 50.1 ± 13.5 years [108], higher plasma spermidine was associated with a lower hippocampal volume, reduced cortical thickness and a higher Alzheimer's disease score. The reason for the discrepancy in the findings is not clear but the latter study's observations are at odds with the beneficial effect of spermidine supplementation in models of neurodegenerative disease. Post‐mortem studies of Alzheimer's disease brain tissue showed that spermidine concentration is significantly elevated in temporal cortex but not other brain regions [109]. In another study, spermidine concentration was found to be higher in frontal and parietal but not occipital cortex of subjects with Alzheimer's disease [110], suggesting that the relationship between spermidine concentration in bodily tissues and fluids and disease is at present unclear and further studies are needed.

The concentration of spermidine in the post‐mortem brain of patients with lysosomal storage disorders has not been studied to the best of our knowledge, however significantly elevated levels of intracellular spermidine were reported in an MPS IIIB cell model, but differences were not present in 8‐month‐old MPS IIIB mouse whole brain samples [103], nor were they consistently noted here in whole MPS IIIA or MPS IIIC flies. The related polyamines spermine and putrescine were, however, significantly reduced and elevated (respectively) in MPS IIIB mouse brain. This observation contrasts with the current findings in whole MPS IIIA and MPS IIIC flies, where no genotype‐related elevation in putrescine levels was noted. Spermine was not quantified in the present metabolomic evaluation. Finally, significantly increased levels of spermidine were observed in MPS I patient CSF samples (c.f. control patient CSF), in a metabolomics evaluation [111]. The data shown in Figure S1 were extracted from the Supporting Information in the study by Hinderer and colleagues [111]. Collectively, the above observations suggest that aberrant polyamine biogenesis, absorption, intracellular trafficking or elimination *may* occur in MPS III, although further ex vivo metabolomic brain studies across multiple MPS III subtypes and model systems are required to confirm it. Future metabolomic analyses in flies should utilise whole heads, rather than whole flies, to limit the observations to brain tissue.

In conclusion, whilst some beneficial outcomes on disease parameters were noted here, additional studies are needed to clarify if and how polyamine homeostasis is altered in MPS III and other related lysosomal storage disorders and whether spermidine supplementation represents a safe, clinical disease‐modifying nutraceutical approach for this and other related disease states. Additional studies are also required to determine whether myo‐inositol levels are altered in patients with MPS III and whether deficiency contributes to brain structure/function abnormalities. If so, myo‐inositol supplementation may be a cheap and widely available strategy for slowing disease progression in this presently untreatable disorder.

## Author Contributions

**Helen Beard:** investigation (tissue processing and immunohistochemistry, mouse studies), formal analysis, writing (review and editing). **Sonia Dayan:** investigation (*Drosophila* behavioural studies), formal analysis, writing (review and editing). **Karissa Barthelson:** investigation (tissue preparation and western blots, mouse studies, metabolomics dataset pre‐processing, abundance and enrichment analysis and data curation), formal analysis, writing (review and editing). **Laura Hewson:** investigation (MPS IIIA and MPS IIIC fly propagation for metabolomics), writing (review and editing). **Louise V. O'Keefe:** conceptualisation of the *Drosophila* studies, resources (MPS IIIA and MPS IIIC *Drosophila* models), formal analysis, data curation, supervision (*Drosophila* studies), funding acquisition (*Drosophila* studies), project administration. **Kim M. Hemsley:** conceptualisation of the mouse studies, resources (MPS IIIA mice), investigation (live mouse studies), formal analysis, data curation, writing (original draft), writing (review and editing), supervision (mouse studies), funding (mouse studies), project administration.

## Funding

This work was supported by Sanfilippo Children's Foundation, Fondation Sanfilippo Suisse and National MPS Society.

## Conflicts of Interest

The authors declare no conflicts of interest.

## Supporting information


**Figure S1:** Quantitation LC3II/LC3I (A) and p62 (B)‐probed western blots (C). Graphs show the mean (bar) with individual mice represented by dots. *p < 0.05. Full size blots were provided for review. The graph in (D) is an evaluation of data in Hinderer et al. [111]. The authors performed metabolomics on unaffected and MPS I patient CSF (*n* = 15 patients/group). There is a statistically significant difference in the amount of spermidine in the MPS I patient CSF c.f. unaffected human CSF (*p* < 0.0001). The paper's supplementary file contains the raw data—https://pmc.ncbi.nlm.nih.gov/articles/instance/5886077/bin/16‐03_hmgr1_suptable1_062917_vf_ddx277.xlsx.

**File S1:** Output from limma differential expression analysis.

**File S2:** Output from fry enrichment analysis.

## Data Availability

The data that support the findings of this study are available from the corresponding author upon reasonable request. The metabolomics data has been deposited in a publicly available repository. The raw metabolomics data is available from the Metabolomics Workbench ST004530. All code to reproduce the metabolomics analysis can be found at https://github.com/karissa‐b/2025_Drosophila_MPSIII_metabolomics.

## References

[1] H. Kresse , “Mucopolysaccharidosis 3 A (Sanfilippo A Disease): Deficiency of a Heparin Sulfamidase in Skin Fibroblasts and Leucocytes,” Biochemical and Biophysical Research Communications 54, no. 3 (1973): 1111–1118. 4201808 10.1016/0006-291x(73)90807-3

[2] H. S. Scott , L. Blanch , X. H. Guo , et al., “Cloning of the Sulphamidase Gene and Identification of Mutations in Sanfilippo A Syndrome,” Nature Genetics 11, no. 4 (1995): 465–467. 7493035 10.1038/ng1295-465

[3] K. von Figura and H. Kresse , “The Sanfilippo B Corrective Factor: A N‐Acetyl‐Alpha‐D‐Glucosamindiase,” Biochemical and Biophysical Research Communications 48, no. 2 (1972): 262–269. 4261365 10.1016/s0006-291x(72)80044-5

[4] H. G. Zhao , H. H. Li , G. Bach , A. Schmidtchen , and E. F. Neufeld , “The Molecular Basis of Sanfilippo Syndrome Type B,” Proceedings of the National Academy of Sciences of the United States of America 93, no. 12 (1996): 6101–6105. 8650226 10.1073/pnas.93.12.6101 PMC39196

[5] U. Klein , H. Kresse , and K. von Figura , “Sanfilippo Syndrome Type C: Deficiency of Acetyl‐CoA:Alpha‐Glucosaminide N‐Acetyltransferase in Skin Fibroblasts,” Proceedings of the National Academy of Sciences of the United States of America 75, no. 10 (1978): 5185–5189. 33384 10.1073/pnas.75.10.5185 PMC336290

[6] X. Fan , H. Zhang , S. Zhang , et al., “Identification of the Gene Encoding the Enzyme Deficient in Mucopolysaccharidosis IIIC (Sanfilippo Disease Type C),” American Journal of Human Genetics 79, no. 4 (2006): 738–744. 16960811 10.1086/508068 PMC1592569

[7] M. Hrebícek , L. Mrázová , V. Seyrantepe , et al., “Mutations in TMEM76* Cause Mucopolysaccharidosis IIIC (Sanfilippo C Syndrome),” American Journal of Human Genetics 79, no. 5 (2006): 807–819. 17033958 10.1086/508294 PMC1698556

[8] H. Kresse , E. Paschke , K. von Figura , W. Gilberg , and W. Fuchs , “Sanfilippo Disease Type D: Deficiency of N‐Acetylglucosamine‐6‐Sulfate Sulfatase Required for Heparan Sulfate Degradation,” Proceedings of the National Academy of Sciences of the United States of America 77, no. 11 (1980): 6822–6826. 6450420 10.1073/pnas.77.11.6822 PMC350382

[9] D. A. Robertson , C. Freeman , C. P. Morris , et al., “A cDNA Clone for Human Glucosamine‐6‐Sulphatase Reveals Differences Between Arylsulphatases and Non‐Arylsulphatases,” Biochemical Journal 288, no. Pt 2 (1992): 539–544. 1463457 10.1042/bj2880539 PMC1132044

[10] S. J. Chin and M. Fuller , “Prevalence of Lysosomal Storage Disorders in Australia From 2009 to 2020,” Lancet Regional Health ‐ Western Pacific 19 (2022): 100344. 35024668 10.1016/j.lanwpc.2021.100344 PMC8671750

[11] H. Y. Lin , S. P. Lin , C. K. Chuang , et al., “Incidence of the Mucopolysaccharidoses in Taiwan, 1984–2004,” American Journal of Medical Genetics. Part A 149A, no. 5 (2009): 960–964. 19396827 10.1002/ajmg.a.32781

[12] S. A. Khan , H. Peracha , D. Ballhausen , et al., “Epidemiology of Mucopolysaccharidoses,” Molecular Genetics and Metabolism 121, no. 3 (2017): 227–240. 28595941 10.1016/j.ymgme.2017.05.016 PMC5653283

[13] C. Ceuterick , J. J. Martin , J. Libert , and J. P. Farriaux , “Sanfilippo A Disease in the Fetus—Comparison With Pre‐ and Postnatal Cases,” Neuropediatrics 11, no. 2 (1980): 176–185. 10.1055/s-2008-1071387 6777713

[14] F. A. Wijburg , K. Aiach , A. Chakrapani , et al., “An Observational, Prospective, Multicenter, Natural History Study of Patients With Mucopolysaccharidosis Type IIIA,” Molecular Genetics and Metabolism 135, no. 2 (2022): 133–142. 34991944 10.1016/j.ymgme.2021.12.002

[15] M. S. Kim , A. Yang , E. S. Noh , et al., “Natural History and Molecular Characteristics of Korean Patients With Mucopolysaccharidosis Type III,” Journal of Personalized Medicine 12, no. 5 (2022): 665. 35629088 10.3390/jpm12050665 PMC9145712

[16] G. J. Ruijter , M. J. Valstar , J. M. van de Kamp , et al., “Clinical and Genetic Spectrum of Sanfilippo Type C (MPS IIIC) Disease in The Netherlands,” Molecular Genetics and Metabolism 93, no. 2 (2008): 104–111. 18024218 10.1016/j.ymgme.2007.09.011

[17] C. Martins , P. F. V. de Medeiros , S. Leistner‐Segal , et al., “Molecular Characterization of a Large Group of Mucopolysaccharidosis Type IIIC Patients Reveals the Evolutionary History of the Disease,” Human Mutation 40, no. 8 (2019): 1084–1100. 31228227 10.1002/humu.23752

[18] E. Shapiro , K. King , A. Ahmed , et al., “The Neurobehavioral Phenotype in Mucopolysaccharidosis Type IIIB: An Exploratory Study,” Molecular Genetics and Metabolism Reports 6 (2016): 41–47. 26918231 10.1016/j.ymgmr.2016.01.003 PMC4762067

[19] K. V. Truxal , H. Fu , D. M. McCarty , et al., “A Prospective One‐Year Natural History Study of Mucopolysaccharidosis Types IIIA and IIIB: Implications for Clinical Trial Design,” Molecular Genetics and Metabolism 119, no. 3 (2016): 239–248. 27590925 10.1016/j.ymgme.2016.08.002

[20] N. J. Abreu , B. Selvaraj , K. V. Truxal , et al., “Longitudinal MRI Brain Volume Changes Over One Year in Children With Mucopolysaccharidosis Types IIIA and IIIB,” Molecular Genetics and Metabolism 133, no. 2 (2021): 193–200. 33962822 10.1016/j.ymgme.2021.04.006

[21] D. A. do Valle , M. L. S. F. Santos , B. A. Telles , and M. L. Cordeiro , “Neurological, Neurobehavioral, and Radiological Alterations in Patients With Mucopolysaccharidosis III (Sanfilippo's Syndrome) in Brazil,” Frontiers in Neurology 13 (2022): 968297. 36468061 10.3389/fneur.2022.968297 PMC9714604

[22] S. Huang , Z. J. Beatty , A. M. Mckinney , and D. R. Nascene , “Increased Pituitary Volumes in Patients With Sanfilippo Syndrome (Mucopolysaccharidosis Type 3, MPS III),” Neuroradiology 65, no. 9 (2023): 1381–1386. 37127720 10.1007/s00234-023-03157-2

[23] P. Anikiej‐Wiczenbach , M. Limanówka , M. Mazurkiewicz‐Bełdzińska , et al., “Development and Longitudinal Neurocognitive Functioning in Mucopolysaccharidosis Type IIIC: A Case Study,” Journal of Applied Genetics 66 (2024): 647–652, 10.1007/s13353-024-00934-4. 39739235

[24] V. Delgadillo , M. M. O'Callaghan , L. Gort , M. J. Coll , and M. Pineda , “Natural History of Sanfilippo Syndrome in Spain,” Orphanet Journal of Rare Diseases 8 (2013): 189. 24314109 10.1186/1750-1172-8-189 PMC3879199

[25] D. L. Webber , A. Choo , L. J. Hewson , et al., “Neuronal‐Specific Impairment of Heparan Sulfate Degradation in Drosophila Reveals Pathogenic Mechanisms for Mucopolysaccharidosis Type IIIA,” Experimental Neurology 303 (2018): 38–47. 29408731 10.1016/j.expneurol.2018.01.020

[26] L. Hewson , A. Choo , D. L. Webber , et al., “ *Drosophila melanogaster* Models of MPS IIIC (Hgsnat‐Deficiency) Highlight the Role of Glia in Disease Presentation,” Journal of Inherited Metabolic Disease 47, no. 2 (2024): 340–354. 38238109 10.1002/jimd.12712

[27] B. Simkhada , N. O. Nazario‐Yepiz , P. S. Freymuth , et al., “A Drosophila Model of Mucopolysaccharidosis IIIB,” Genetics 229, no. 3 (2025): iyae219. 39737777 10.1093/genetics/iyae219 PMC11912869

[28] A. M. Douek , M. Amiri Khabooshan , J. Henry , et al., “An Engineered Sgsh Mutant Zebrafish Recapitulates Molecular and Behavioural Pathobiology of Sanfilippo Syndrome A/MPS IIIA,” International Journal of Molecular Sciences 22, no. 11 (2021): 5948. 34073041 10.3390/ijms22115948 PMC8197930

[29] E. Gerken , S. Ahmad , L. Rattan , K. Hemsley , K. Barthelson , and M. Lardelli , “Zebrafish Models of Mucopolysaccharidosis Types IIIA, B, & C Show Hyperactivity and Changes in Oligodendrocyte State,” bioRxiv, 10.1101/2023.08.02.550904.

[30] K. Barthelson , R. A. Protzman , M. F. Snel , K. Hemsley , and M. Lardelli , “Multi‐Omics Analyses of Early‐Onset Familial Alzheimer's Disease and Sanfilippo Syndrome Zebrafish Models Reveal Commonalities in Disease Mechanisms,” Biochimica et Biophysica Acta ‐ Molecular Basis of Disease 1871, no. 3 (2025): 167651. 39798820 10.1016/j.bbadis.2024.167651

[31] H. H. Li , W. H. Yu , N. Rozengurt , et al., “Mouse Model of Sanfilippo Syndrome Type B Produced by Targeted Disruption of the Gene Encoding Alpha‐N‐Acetylglucosaminidase,” Proceedings of the National Academy of Sciences of the United States of America 96, no. 25 (1999): 14505–14510. 10588735 10.1073/pnas.96.25.14505 PMC24466

[32] M. Bhaumik , V. J. Muller , T. Rozaklis , et al., “A Mouse Model for Mucopolysaccharidosis Type III A (Sanfilippo Syndrome),” Glycobiology 9, no. 12 (1999): 1389–1396. 10561464 10.1093/glycob/9.12.1389

[33] C. Martins , H. Hůlková , L. Dridi , et al., “Neuroinflammation, Mitochondrial Defects and Neurodegeneration in Mucopolysaccharidosis III Type C Mouse Model,” Brain 138, no. Pt 2 (2015): 336–355. 25567323 10.1093/brain/awu355 PMC4306821

[34] C. Roca , S. Motas , S. Marcó , et al., “Disease Correction by AAV‐Mediated Gene Therapy in a New Mouse Model of Mucopolysaccharidosis Type IIID,” Human Molecular Genetics 26, no. 8 (2017): 1535–1551. 28334745 10.1093/hmg/ddx058

[35] E. L. Aronovich , J. M. Johnston , P. Wang , U. Giger , and C. B. Whitley , “Molecular Basis of Mucopolysaccharidosis Type IIIB in Emu ( *Dromaius novaehollandiae* ): An Avian Model of Sanfilippo Syndrome Type B,” Genomics 74, no. 3 (2001): 299–305. 11414757 10.1006/geno.2001.6552

[36] J. N. Thompson , M. Z. Jones , G. Dawson , and P. S. Huffman , “N‐Acetylglucosamine 6‐Sulphatase Deficiency in a Nubian Goat: A Model of Sanfilippo Syndrome Type D (Mucopolysaccharidosis IIID),” Journal of Inherited Metabolic Disease 15, no. 5 (1992): 760–768. 1434515 10.1007/BF01800018

[37] Q. Yang , X. Zhao , Y. Xing , et al., “A Model of Mucopolysaccharidosis Type IIIB in Pigs,” Biology Open 7, no. 10 (2018): bio035386. 30257828 10.1242/bio.035386 PMC6215415

[38] L. Karageorgos , B. Hill , M. J. Bawden , and J. J. Hopwood , “Bovine Mucopolysaccharidosis Type IIIB,” Journal of Inherited Metabolic Disease 30, no. 3 (2007): 358–364. 17458708 10.1007/s10545-007-0539-5

[39] A. Fischer , K. P. Carmichael , J. F. Munnell , et al., “Sulfamidase Deficiency in a Family of Dachshunds: A Canine Model of Mucopolysaccharidosis IIIA (Sanfilippo A),” Pediatric Research 44, no. 1 (1998): 74–82. 9667374 10.1203/00006450-199807000-00012

[40] G. Yogalingam , T. Pollard , B. Gliddon , R. D. Jolly , and J. J. Hopwood , “Identification of a Mutation Causing Mucopolysaccharidosis Type IIIA in New Zealand Huntaway Dogs,” Genomics 79, no. 2 (2002): 150–153. 11829484 10.1006/geno.2002.6699

[41] N. M. Ellinwood , P. Wang , T. Skeen , et al., “A Model of Mucopolysaccharidosis IIIB (Sanfilippo Syndrome Type IIIB): N‐Acetyl‐Alpha‐D‐Glucosaminidase Deficiency in Schipperke Dogs,” Journal of Inherited Metabolic Disease 26, no. 5 (2003): 489–504. 14518829 10.1023/a:1025177411938

[42] R. McGlynn , K. Dobrenis , and S. U. Walkley , “Differential Subcellular Localization of Cholesterol, Gangliosides, and Glycosaminoglycans in Murine Models of Mucopolysaccharide Storage Disorders,” Journal of Comparative Neurology 480, no. 4 (2004): 415–426. 15558784 10.1002/cne.20355

[43] G. M. Viana , D. A. Priestman , F. M. Platt , S. Khan , S. Tomatsu , and A. V. Pshezhetsky , “Brain Pathology in Mucopolysaccharidoses (MPS) Patients With Neurological Forms,” Journal of Clinical Medicine 9, no. 2 (2020): 396. 32024172 10.3390/jcm9020396 PMC7073982

[44] A. A. Lau , P. J. Trim , B. M. King , et al., “Filipin Complex‐Reactive Brain Lesions: A Cautionary Tale,” Neuropathology and Applied Neurobiology 50, no. 1 (2024): e12950. 10.1111/nan.12950 38112248

[45] H. Beard , S. Hassiotis , W. P. Gai , E. Parkinson‐Lawrence , J. J. Hopwood , and K. M. Hemsley , “Axonal Dystrophy in the Brain of Mice With Sanfilippo Syndrome,” Experimental Neurology 295 (2017): 243–255. 28601604 10.1016/j.expneurol.2017.06.010

[46] C. Settembre , A. Fraldi , L. Jahreiss , et al., “A Block of Autophagy in Lysosomal Storage Disorders,” Human Molecular Genetics 17, no. 1 (2008): 119–129. 17913701 10.1093/hmg/ddm289

[47] A. Arfi , M. Richard , C. Gandolphe , D. Bonnefont‐Rousselot , P. Thérond , and D. Scherman , “Neuroinflammatory and Oxidative Stress Phenomena in MPS IIIA Mouse Model: The Positive Effect of Long‐Term Aspirin Treatment,” Molecular Genetics and Metabolism 103, no. 1 (2011): 18–25. 21353610 10.1016/j.ymgme.2011.01.015

[48] D. A. Salazar , A. Rodríguez‐López , A. Herreño , et al., “Systems Biology Study of Mucopolysaccharidosis Using a Human Metabolic Reconstruction Network,” Molecular Genetics and Metabolism 117, no. 2 (2016): 129–139. 26276570 10.1016/j.ymgme.2015.08.001

[49] C. Pará , P. Bose , L. Bruno , et al., “Early Defects in Mucopolysaccharidosis Type IIIC Disrupt Excitatory Synaptic Transmission,” JCI Insight 6, no. 15 (2021): e142073. 34156977 10.1172/jci.insight.142073 PMC8410035

[50] J. DiRosario , E. Divers , C. Wang , et al., “Innate and Adaptive Immune Activation in the Brain of MPS IIIB Mouse Model,” Journal of Neuroscience Research 87, no. 4 (2009): 978–990. 18951493 10.1002/jnr.21912

[51] S. Hassiotis , H. Beard , A. Luck , et al., “Disease Stage Determines the Efficacy of Treatment of a Paediatric Neurodegenerative Disease,” European Journal of Neuroscience 39, no. 12 (2014): 2139–2150. 25068161 10.1111/ejn.12557

[52] M. Taherzadeh , E. Zhang , I. Londono , et al., “Severe Central Nervous System Demyelination in Sanfilippo Disease,” Frontiers in Molecular Neuroscience 16 (2023): 1323449. 38163061 10.3389/fnmol.2023.1323449 PMC10756675

[53] I. Sambri , R. D'Alessio , Y. Ezhova , et al., “Lysosomal Dysfunction Disrupts Presynaptic Maintenance and Restoration of Presynaptic Function Prevents Neurodegeneration in Lysosomal Storage Diseases,” EMBO Molecular Medicine 9, no. 1 (2017): 112–132. 27881461 10.15252/emmm.201606965 PMC5210158

[54] C. B. P. de Aragão , L. Bruno , P. Bose , et al., “Early Defects in Lysosomal Storage Diseases Disrupt Excitatory Synaptic Transmission,” bioRxiv, 10.1101/2020.07.06.186809.

[55] K. M. Hemsley , B. King , and J. J. Hopwood , “Injection of Recombinant Human Sulfamidase Into the CSF via the Cerebellomedullary Cistern in MPS IIIA Mice,” Molecular Genetics and Metabolism 90, no. 3 (2007): 313–328. 17166757 10.1016/j.ymgme.2006.10.005

[56] M. Malinowska , F. L. Wilkinson , K. J. Langford‐Smith , et al., “Genistein Improves Neuropathology and Corrects Behaviour in a Mouse Model of Neurodegenerative Metabolic Disease,” PLoS One 5, no. 12 (2010): e14192. 21152017 10.1371/journal.pone.0014192 PMC2995736

[57] N. M. Ellinwood , J. Ausseil , N. Desmaris , et al., “Safe, Efficient, and Reproducible Gene Therapy of the Brain in the Dog Models of Sanfilippo and Hurler Syndromes,” Molecular Therapy 19, no. 2 (2011): 251–259. 21139569 10.1038/mt.2010.265 PMC3034858

[58] A. Ruzo , S. Marcó , M. García , et al., “Correction of Pathological Accumulation of Glycosaminoglycans in Central Nervous System and Peripheral Tissues of MPSIIIA Mice Through Systemic AAV9 Gene Transfer,” Human Gene Therapy 23, no. 12 (2012): 1237–1246. 22909060 10.1089/hum.2012.029

[59] V. Haurigot , S. Marcó , A. Ribera , et al., “Whole Body Correction of Mucopolysaccharidosis IIIA by Intracerebrospinal Fluid Gene Therapy,” Journal of Clinical Investigation 123, no. 8 (2013): 3254–3271. 23863627 10.1172/JCI66778 PMC3726158

[60] A. Sergijenko , A. Langford‐Smith , A. Y. Liao , et al., “Myeloid/Microglial Driven Autologous Hematopoietic Stem Cell Gene Therapy Corrects a Neuronopathic Lysosomal Disease,” Molecular Therapy 21, no. 10 (2013): 1938–1949. 23748415 10.1038/mt.2013.141 PMC3808137

[61] B. King , N. Marshall , H. Beard , et al., “Evaluation of Enzyme Dose and Dose‐Frequency in Ameliorating Substrate Accumulation in MPS IIIA Huntaway Dog Brain,” Journal of Inherited Metabolic Disease 38, no. 2 (2015): 341–350. 25421091 10.1007/s10545-014-9790-8

[62] L. K. Winner , H. Beard , S. Hassiotis , et al., “A Preclinical Study Evaluating AAVrh10‐Based Gene Therapy for Sanfilippo Syndrome,” Human Gene Therapy 27, no. 5 (2016): 363–375. 26975339 10.1089/hum.2015.170

[63] H. Fu , M. P. Cataldi , T. A. Ware , et al., “Functional Correction of Neurological and Somatic Disorders at Later Stages of Disease in MPS IIIA Mice by Systemic scAAV9‐hSGSH Gene Delivery,” Molecular Therapy ‐ Methods and Clinical Development 3 (2016): 16036. 27331076 10.1038/mtm.2016.36 PMC4898406

[64] P. Lotfi , D. Y. Tse , A. Di Ronza , et al., “Trehalose Reduces Retinal Degeneration, Neuroinflammation and Storage Burden Caused by a Lysosomal Hydrolase Deficiency,” Autophagy 14, no. 8 (2018): 1419–1434. 29916295 10.1080/15548627.2018.1474313 PMC6103706

[65] M. Hocquemiller , K. M. Hemsley , and M. L. Douglass , “AAVrh10 Vector Corrects Disease Pathology in MPS IIIA Mice and Achieves Widespread Distribution of SGSH in Large Animal Brains,” Molecular Therapy—Methods and Clinical Development 17 (2019): 174–187. 31909089 10.1016/j.omtm.2019.12.001 PMC6940615

[66] H. Parker , S. M. Ellison , R. J. Holley , et al., “Haematopoietic Stem Cell Gene Therapy With IL‐1Ra Rescues Cognitive Loss in Mucopolysaccharidosis IIIA,” EMBO Molecular Medicine 12, no. 3 (2020): e11185. 32057196 10.15252/emmm.201911185 PMC7059006

[67] N. M. Ellinwood , B. N. Valentine , A. S. Hess , et al., “Tralesinidase Alfa Enzyme Replacement Therapy Prevents Disease Manifestations in a Canine Model of Mucopolysaccharidosis Type IIIB,” Journal of Pharmacology and Experimental Therapeutics 382, no. 3 (2022): 277–286. 35717448 10.1124/jpet.122.001119 PMC9426762

[68] E. Rintz , M. Podlacha , Z. Cyske , K. Pierzynowska , G. Węgrzyn , and L. Gaffke , “Activities of (Poly)phenolic Antioxidants and Other Natural Autophagy Modulators in the Treatment of Sanfilippo Disease: Remarkable Efficacy of Resveratrol in Cellular and Animal Models,” Neurotherapeutics 20, no. 1 (2023): 254–271. 36344724 10.1007/s13311-022-01323-7 PMC10119361

[69] X. Pan , A. Caillon , S. Fan , S. Khan , S. Tomatsu , and A. V. Pshezhetsky , “Heterologous HSPC Transplantation Rescues Neuroinflammation and Ameliorates Peripheral Manifestations in the Mouse Model of Lysosomal Transmembrane Enzyme Deficiency, MPS IIIC,” Cells 13, no. 10 (2024): 877. 38786099 10.3390/cells13100877 PMC11120110

[70] H. Wei , Z. Zhang , A. Saha , et al., “Disruption of Adaptive Energy Metabolism and Elevated Ribosomal p‐S6K1 Levels Contribute to INCL Pathogenesis: Partial Rescue by Resveratrol,” Human Molecular Genetics 20, no. 6 (2011): 1111–1121. 21224254 10.1093/hmg/ddq555 PMC3043662

[71] M. Mirza , C. Volz , M. Karlstetter , et al., “Progressive Retinal Degeneration and Glial Activation in the CLN6 (Nclf) Mouse Model of Neuronal Ceroid Lipofuscinosis: A Beneficial Effect of DHA and Curcumin Supplementation,” PLoS One 8, no. 10 (2013): e75963. 24124525 10.1371/journal.pone.0075963 PMC3790850

[72] S. Bar , M. Prasad , and R. Datta , “Neuromuscular Degeneration and Locomotor Deficit in a *Drosophila* Model of Mucopolysaccharidosis VII Is Attenuated by Treatment With Resveratrol,” Disease Models and Mechanisms 11, no. 11 (2018): dmm036954. 30459155 10.1242/dmm.036954 PMC6262814

[73] M. Mobini , S. Radbakhsh , F. Kubaski , et al., “Impact of Intravenous Trehalose Administration in Patients With Niemann‐Pick Disease Types A and B,” Journal of Clinical Medicine 11, no. 1 (2022): 247. 35011993 10.3390/jcm11010247 PMC8745869

[74] M. Mobini , S. Radbakhsh , F. Kubaski , et al., “Effects of Trehalose Administration in Patients With Mucopolysaccharidosis Type III,” Current Medicinal Chemistry 31, no. 20 (2024): 3033–3042. 37038706 10.2174/0929867330666230406102555

[75] T. Eisenberg , M. Abdellatif , S. Schroeder , et al., “Cardioprotection and Lifespan Extension by the Natural Polyamine Spermidine,” Nature Medicine 22, no. 12 (2016): 1428–1438. 10.1038/nm.4222 PMC5806691 27841876

[76] K. Freitag , N. Sterczyk , S. Wendlinger , et al., “Spermidine Reduces Neuroinflammation and Soluble Amyloid Beta in an Alzheimer's Disease Mouse Model,” Journal of Neuroinflammation 19, no. 1 (2022): 172. 35780157 10.1186/s12974-022-02534-7 PMC9250727

[77] D. Lumkwana , C. Peddie , J. Kriel , et al., “Investigating the Role of Spermidine in a Model System of Alzheimer's Disease Using Correlative Microscopy and Super‐Resolution Techniques,” Frontiers in Cell and Development Biology 10 (2022): 819571. 10.3389/fcell.2022.819571 PMC9152225 35656544

[78] S. Sharma , P. Kumar , and R. Deshmukh , “Neuroprotective Potential of Spermidine Against Rotenone Induced Parkinson's Disease in Rats,” Neurochemistry International 116 (2018): 104–111. 29501454 10.1016/j.neuint.2018.02.010

[79] S. Kiechl , R. Pechlaner , P. Willeit , et al., “Higher Spermidine Intake Is Linked to Lower Mortality: A Prospective Population‐Based Study,” American Journal of Clinical Nutrition 108, no. 2 (2018): 371–380. 29955838 10.1093/ajcn/nqy102

[80] M. E. Ritchie , B. Phipson , D. Wu , et al., “Limma Powers Differential Expression Analyses for RNA‐Sequencing and Microarray Studies,” Nucleic Acids Research 43, no. 7 (2015): e47. 25605792 10.1093/nar/gkv007 PMC4402510

[81] Y. Benjamini and Y. Hochberg , “Controlling the False Discovery Rate: A Practical and Powerful Approach to Multiple Testing,” Journal of the Royal Statistical Society: Series B: Methodological 57, no. 1 (1995): 289–300.

[82] D. Wu , E. Lim , F. Vaillant , M. L. Asselin‐Labat , J. E. Visvader , and G. K. Smyth , “ROAST: Rotation Gene Set Tests for Complex Microarray Experiments,” Bioinformatics 26, no. 17 (2010): 2176–2182. 20610611 10.1093/bioinformatics/btq401 PMC2922896

[83] M. Kanehisa , M. Furumichi , Y. Sato , et al., “KEGG: Biological Systems Database as a Model of the Real World,” Nucleic Acids Research 53, no. D1 (2025): D672–D677. 39417505 10.1093/nar/gkae909 PMC11701520

[84] A. A. Lau , N. J. Shamsani , L. K. Winner , et al., “Neonatal Bone Marrow Transplantation in MPS IIIA Mice,” JIMD Reports 8 (2013): 121–132. 23430528 10.1007/8904_2012_169 PMC3565653

[85] H. Beard , G. Chidlow , D. Neumann , et al., “Is the Eye a Window to the Brain in Sanfilippo Syndrome?,” Acta Neuropathologica Communications 8, no. 1 (2020): 194. 33203474 10.1186/s40478-020-01070-w PMC7672954

[86] S. L. Tan , D. Neumann , P. J. Trim , et al., “Substrate Reduction Using a Glucosamine Analogue in Drosophila Melanogaster and Mouse Models of Sanfilippo Syndrome,” Molecular Genetics and Metabolism 145, no. 2 (2025): 109112. 40288156 10.1016/j.ymgme.2025.109112

[87] S. Schroeder , S. J. Hoffer , A. Zimmermann , et al., “Dietary Spermidine Improves Cognitive Function,” Cell Reports 35, no. 2 (2021): 10898. 10.1016/j.celrep.2021.108985 33852843

[88] H. Fu , A. S. Meadows , T. Ware , R. P. Mohney , and D. M. McCarty , “Near‐Complete Correction of Profound Metabolomic Impairments Corresponding to Functional Benefit in MPS IIIB Mice After IV rAAV9‐hNAGLU Gene Delivery,” Molecular Therapy 25, no. 3 (2017): 792–802. 28143737 10.1016/j.ymthe.2016.12.025 PMC5363204

[89] H. Fu , A. S. Meadows , R. J. Pineda , R. P. Mohney , S. Stirdivant , and D. M. McCarty , “Serum Global Metabolomics Profiling Reveals Profound Metabolic Impairments in Patients With MPS IIIA and MPS IIIB,” Metabolic Brain Disease 32 (2017): 1403–1415. 28382573 10.1007/s11011-017-0009-1

[90] E. Niess , F. Niess , W. Bogner , et al., “Myo‐Inositol Deficiency, Structural Brain Changes, and Cerebral Perfusion Alterations in Classic Galactosemia: Preliminary Insights From a Multiparametric MRI Study,” Journal of Inherited Metabolic Disease 48, no. 6 (2025): e70097. 41083920 10.1002/jimd.70097 PMC12518697

[91] S. Hagen‐Lillevik , J. Johnson , A. Siddiqi , J. Persinger , G. Hale , and K. Lai , “Harnessing the Power of Purple Sweet Potato Color and Myo‐Inositol to Treat Classic Galactosemia,” International Journal of Molecular Sciences 23, no. 15 (2022): 8654. 35955788 10.3390/ijms23158654 PMC9369367

[92] O. Bellagamba , A. J. Guo , S. Senthilkumar , et al., “Assessment of Long‐Term Safety and Efficacy of Purple Sweet Potato Color (PSPC) and Myo‐Inositol (MI) Treatment for Motor Related and Behavioral Phenotypes in a Mouse Model of Classic Galactosemia,” Journal of Inherited Metabolic Disease 48, no. 2 (2025): e70002. 39894675 10.1002/jimd.70002 PMC11788002

[93] M. Zuccarini , L. Pruccoli , M. Balducci , et al., “Influence of Guanine‐Based Purines on the Oxidoreductive Reactions Involved in Normal or Altered Brain Functions,” Journal of Clinical Medicine 12, no. 3 (2023): 1172. 36769818 10.3390/jcm12031172 PMC9917437

[94] C. I. Tasca , M. Zuccarini , P. Di Iorio , and F. Ciruela , “Lessons From the Physiological Role of Guanosine in Neurodegeneration and Cancer: Toward a Multimodal Mechanism of Action?,” Purinergic Signal 21, no. 1 (2025): 133–148. 39004650 10.1007/s11302-024-10033-y PMC11958862

[95] R. Das and M. S. Kanungo , “Activity and Modulation of Ornithine Decarboxylase and Concentrations of Polyamines in Various Tissues of Rats as a Function of Age,” Experimental Gerontology 17 (1982): 95–103. 7106211 10.1016/0531-5565(82)90042-0

[96] L. D. Morrison , L. Becker , L. C. Ang , and S. J. Kish , “Polyamines in Human Brain: Regional Distribution and Influence of Aging,” Journal of Neurochemistry 65, no. 2 (1995): 636–642. 7616219 10.1046/j.1471-4159.1995.65020636.x

[97] M. Vivó , N. de Vera , R. Cortés , et al., “Polyamines in the Basal Ganglia of Human Brain. Influence of Aging and Degenerative Movement Disorders,” Neuroscience Letters 304, no. 1–2 (2001): 107–111. 11335066 10.1016/s0304-3940(01)01776-1

[98] C. Dwyer , S. Scudder , Y. Lin , et al., “Neurodevelopmental Changes in Excitatory Synaptic Structure and Function in the Cerebral Cortex of Sanfilippo Syndrome IIIA Mice,” Scientific Reports 7 (2017): 46576. 28418018 10.1038/srep46576 PMC5394534

[99] A. Santos‐Gómez , N. Juliá‐Palacios , A. Rejano‐Bosch , et al., “Spermidine Treatment Improves GRIN2B Loss‐Of‐Function, a Primary Disorder of Glutamatergic Neurotransmission,” Journal of Inherited Metabolic Disease 48, no. 2 (2025): e70015. 40024627 10.1002/jimd.70015 PMC11872566

[100] C. Schwarz , G. S. Benson , N. Horn , et al., “Effects of Spermidine Supplementation on Cognition and Biomarkers in Older Adults With Subjective Cognitive Decline: A Randomized Clinical Trial,” JAMA Network Open 5, no. 5 (2022): e2213875. 35616942 10.1001/jamanetworkopen.2022.13875 PMC9136623

[101] T. Pekar , A. Wendzel , and R. Jarisch , “The Positive Effect of Spermidine in Older Adults Suffering From Dementia After 1 Year,” Wiener Klinische Wochenschrift 136, no. 1–2 (2024): 64–66. 37284840 10.1007/s00508-023-02226-z PMC10776733

[102] T. Hirose , R. Saiki , Y. Yoshizawa , et al., “Spermidine and Ca(2+), but Not Na(+), Can Permeate NMDA Receptors Consisting of GluN1 and GluN2A or GluN2B in the Presence of Mg(2+),” Biochemical and Biophysical Research Communications 463, no. 4 (2015): 1190–1195. 26086092 10.1016/j.bbrc.2015.06.081

[103] M. Scarcella , G. Scerra , M. Ciampa , et al., “Metabolic Rewiring and Autophagy Inhibition Correct Lysosomal Storage Disease in Mucopolysaccharidosis IIIB,” iScience 27, no. 3 (2024): 108959. 38361619 10.1016/j.isci.2024.108959 PMC10864807

[104] G. Liu , H. Y. Chen , X. He , et al., “Adeno‐Associated Virus Type 5 Reduces Learning Deficits and Restores Glutamate Receptor Subunit Levels in MPS VII Mice CNS,” Molecular Therapy 15, no. 2 (2007): 242–247. 17235300 10.1038/sj.mt.6300016

[105] H. M. Wallace , A. V. Fraser , and A. Hughes , “A Perspective of Polyamine Metabolism,” Biochemical Journal 376, no. Pt 1 (2003): 1–14. 13678416 10.1042/BJ20031327 PMC1223767

[106] F. Madeo , S. J. Hofer , T. Pendl , et al., “Nutritional Aspects of Spermidine,” Annual Review of Nutrition 40 (2020): 135–159. 10.1146/annurev-nutr-120419-015419 32634331

[107] T. Pekar , A. Wendzel , W. Flak , et al., “Spermidine in Dementia: Relation to Age and Memory Performance,” Wiener Klinische Wochenschrift 132, no. 1–2 (2020): 42–46. 31832773 10.1007/s00508-019-01588-7 PMC6978435

[108] S. M. Wortha , J. Schulz , J. Hanna , et al., “Association of Spermidine Blood Levels With Microstructure of Sleep‐Implications From a Population‐Based Study,” Geroscience 46, no. 1 (2024): 1319–1330. 37548882 10.1007/s11357-023-00886-3 PMC10828152

[109] L. D. Morrison and S. J. Kish , “Brain Polyamine Levels Are Altered in Alzheimer's Disease,” Neuroscience Letters 197, no. 1 (1995): 5–8. 8545054 10.1016/0304-3940(95)11881-v

[110] K. Inoue , H. Tsutsui , H. Akatsu , et al., “Metabolic Profiling of Alzheimer's Disease Brains,” Scientific Reports 3 (2013): 2364. 23917584 10.1038/srep02364 PMC3734482

[111] C. Hinderer , N. Katz , J. P. Louboutin , et al., “Abnormal Polyamine Metabolism Is Unique to the Neuropathic Forms of MPS: Potential for Biomarker Development and Insight Into Pathogenesis,” Human Molecular Genetics 26, no. 19 (2017): 3837–3849. 28934395 10.1093/hmg/ddx277 PMC5886077

