# Supplementary material for: Short‐Term Oral Spermidine Supplementation Modifies Aspects of Neurodegenerative Disease in Flies and Mice With MPS III
Source: J Inherit Metab Dis. 2026 Apr 28;49:e70195. doi: 10.1002/jimd.70195 (PMC13125739; doi:10.1002/jimd.70195)
Supplement: Supplementary file 1 — Figure S1: Quantitation LC3II/LC3I (A) and p62 (B)‐probed western blots (C). Graphs show the mean (bar) with individual mice represented by dots. *p < 0.05. Full size blots were provided for review. The graph in (D) is an evaluation of data in Hinderer et al. [111]. The authors performed metabolomics on unaffected and MPS I patient CSF (n = 15 patients/group). There is a statistically significant difference in the amount of spermidine in the MPS I patient CSF c.f. unaffected human CSF (p < 0.0001). The paper's supplementary file contains the raw data—https://pmc.ncbi.nlm.nih.gov/articles/instance/5886077/bin/16‐03_hmgr1_suptable1_062917_vf_ddx277.xlsx. [file JIMD-49-0-s002.docx]

**Supplementary Figure 1 -** Quantitation LC3II/LC3I (A) and p62 (B)-probed western blots (C). Graphs show the mean (bar) with individual mice represented by dots. *p<0.05. Full size blots were provided for review. The graph in (D) is an evaluation of data in Hinderer et al (2017). The authors performed metabolomics on unaffected and MPS I patient CSF (n=15 patients/group). There is a statistically significant difference in the amount of spermidine in the MPS I patient CSF c.f. unaffected human CSF (p<0.0001). The paper’s supplementary file contains the raw data - <https://pmc.ncbi.nlm.nih.gov/articles/instance/5886077/bin/16-03_hmgr1_suptable1_062917_vf_ddx277.xlsx> .

**D**
